# How Metabolomics Provides Novel Insights on Celiac Disease and Gluten-Free Diet: A Narrative Review

**DOI:** 10.3389/fmicb.2022.859467

**Published:** 2022-06-23

**Authors:** Mirco Vacca, Annalisa Porrelli, Francesco Maria Calabrese, Tamara Lippolis, Ilaria Iacobellis, Giuseppe Celano, Daniela Pinto, Francesco Russo, Gianluigi Giannelli, Maria De Angelis

**Affiliations:** ^1^Department of Soil, Plant and Food Science, University of Bari Aldo Moro, Bari, Italy; ^2^National Institute of Gastroenterology “S. de Bellis,” Institute of Research, Castellana Grotte, Italy; ^3^Human Microbiome Advanced Project-HMPA, Giuliani SpA, Milan, Italy

**Keywords:** celiac disease, gluten-free diet, metabolomics, biomarkers, dysbiosis, gut microbiota

## Abstract

Celiac disease (CD) is an inflammatory autoimmune disorder triggered by the ingestion of gluten from wheat and other cereals. Nowadays, its positive diagnosis is based on invasive approaches such as the histological examination of intestinal biopsies and positive serology screening of antibodies. After proven diagnosis, the only admissible treatment for CD individuals is strict life-long adherence to gluten-free diet (GFD), although it is not a conclusive therapy. Acting by different mechanisms and with different etiologies, both CD and GFD have a great impact on gut microbiota that result in a different taxa composition. Altered production of specific metabolites reflects these microbiota changes. In this light, the currently available literature reports some suggestions about the possible use of specific metabolites, detected by meta-omics analyses, as potential biomarkers for a CD non-invasive diagnosis. To highlight insights about metabolomics application in CD study, we conducted a narrative dissertation of selected original articles published in the last decade. By applying a systematic search, it clearly emerged how the metabolomic signature appears to be contradictory, as well as poorly investigated.

## Introduction

Celiac disease (CD) is an autoimmune enteropathy triggered by the ingestion of gluten from wheat, rye, barley, oats, spelt, and their cross-related varieties (Schuppan et al., 2009). Based on serologic tests for transglutaminase or anti-endomysial antibodies, the CD prevalence was estimated to account for approximately 1.4%. However, this value reduced to 0.7% based on biopsy-proven screening (Singh et al., 2018). Until the 1970s, three biopsies from the small intestine were required to diagnose CD. Nowadays, a positive serologic test together with a small intestinal biopsy is sufficient to confirm the diagnosis (Singh et al., 2018). Noteworthy, some limitations deserve a deeper clarification. Approximately 3–5% of CD patients are seronegative (Schiepatti et al., 2018). Moreover, villous atrophy was not exclusively detected in CD, therefore, a biopsy can lead to an erroneous diagnosis (Schiepatti et al., 2019). Considering the invasiveness of these methods, novel approaches need to be considered. Flow cytometry is an example of alternative methods applied for CD biomarker assessment (Leon, 2011), as well as the emerging techniques based on the detection of gluten-specific CD4+ cells in peripheral blood mononuclear cells (PBMCs) (Kurki et al., 2021).

While showing symptoms related to diarrhea, lethargy, tiredness with or without anemia, and weight loss (Garrote et al., 2008), CD individuals suffer from various degrees of intestinal inflammation due to the interaction between gluten peptides and intestinal lamina propria (Elli et al., 2015). This leads to the secretion of several proinflammatory cytokines (e.g., IL-1b, IL-8, IL-15, IL-21, TNF-α, and MCP-1) (Schuppan et al., 2009; Iacomino et al., 2016). Specifically, literature reports a chronic upregulation of the IL-15 cytokine, whose increase in the epithelium and intestinal lamina propria is considered a hallmark of mucosal damage (Abadie and Jabri, 2014).

However, other compounds over cytokines are assessed during the pathological progression of many diseases and for this reason, they have been proposed as potential biomarkers of health. Some of them are metabolites related to gut microbiota activity (e.g., free fatty acids – FFAs), and some others might derive from the host. In line with this, evidence suggests that some variations in specific metabolic pathways markedly characterize various human pathological states, including CD (e.g., glycolysis, protein metabolisms, and lipids synthesis) (Caio et al., 2019). In individuals with overt CD, the gluten-free diet (GFD) is the only therapeutic approach currently available to reduce symptoms, villus atrophy, and restore intestinal functionality (Aljada et al., 2021). Although ameliorating CD individual well-being, GFD is not conclusive. Strict adherence to GFD is not easy to be followed also considering that gluten traces have been detected in many processed foods (Olshan et al., 2020). Furthermore, due to persisting symptomatology, malabsorption, and villus atrophy, clinical data showed that a percentage of CD individuals is composed of GFD non-responders. This condition is also known as refractory CD (RCD) and up to date no targeted therapies have been developed to treat them. In line with this, evidence supports that the degree of gluten sensitivity cannot be standardized worldwide due to a large interindividual variability, reflecting the combination of multiple co-factors such as genetic, immune, eating habits, and microbiota involvements (Kivelä et al., 2021). Additionally, GFD requires the replacement of wheat flour with other types of cereals and pseudocereals that usually are characterized by a higher glycemic index, such as corn and rice flours. Considering that diet is one of the major drivers of human gut microbiota composition (Marasco et al., 2016), it is reasonable to think that GFD can directly affect the host’s physiology and metabolism (Carroccio et al., 2021), as well as the gut bacterial activity (Bonder et al., 2016). For this reason, the interactions between the host’s genetics, GFD, and microbiota need to be deepened.

To investigate the metabolites associated with diagnosis and progression of CD (treated or not with a GFD), the present narrative review, based on a systematic search, argues on specific studies published in the 2009–2020 time window. Our primary aim was to provide a critical overview of the main metabolomic variations linked to the interaction between host, gut microbiota, and diet. This inspection offers an additional point of view about the chance of exploiting some specific metabolites as pathology biomarkers.

## Materials and Methods

### Search Strategy and Study Selection

**FIGURE 1:**
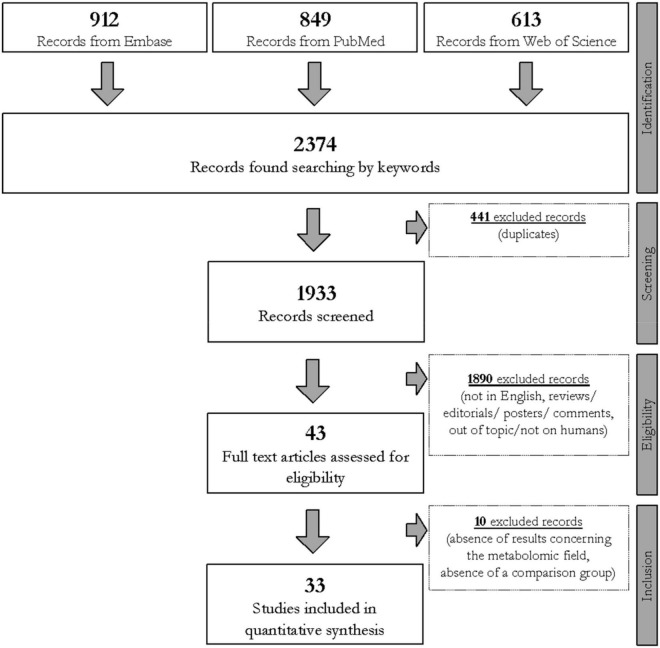
Flowchart of the study selection process carried out using Preferred Reporting Items for Systematic Reviews and Meta-Analyses (PRISMA) process (Shamseer et al., 2015) based on the query line: *(celiac OR coeliac) AND “disease” AND (“metabolomic” OR “metabonomic” OR “metabolome” OR “metabolite” OR “biomarker”) AND (“gluten” OR “diet”)*.

Three electronic databases, specifically PubMed, Embase, and Web of Science, were queried to collect articles in which metabolomic techniques were applied to profile subjects affected by CD. Specific words (i.e., “celiac” OR “coeliac” disease, “metabolomics,” “metabonomics,” “metabolome,” “metabolites,” “biomarkers,” “gluten,” and “diet”) were merged and differently combined with the use of Boolean operators to query the databases. The search was restricted to articles dated between January 2009 and January 2021, agreeing with the eligibility and inclusion criteria consistent with the scope of this narrative review. The search and the selection process were summarized in the step-by-step workflow reported in Figure 1.

### Study Selection

Three independent investigators (AP, MV, and TL) manually evaluated each one of the collected articles after the removal of duplicates. The selection involved all research articles written in English, in which metabolomics was applied to profile human biological samples collected from CD subjects. Only case–control, cross-sectional, and longitudinal studies were considered. Reviews and meta-analyses were excluded. There was no limitation to either the size cohort or the subject’s age. No exclusion criteria involving gender existed. *In vitro* studies, studies on animals or models, and human studies concerning gluten sensibility without a positive diagnosis of CD were discarded. Additionally, to reduce biases, with the only exception of GFD treatment, interventional studies in which the enrolled subjects followed a dietary treatment with probiotics, prebiotics, or synbiotics were also excluded. Hence, 33 original studies were finally selected and summarized in Table 1.

**TABLE 1:** Summary of metabolite variations for different types of biological samples in selected studies.

Groups	Sample	Investigative methodology	Levels of metabolites		References
			↑	↓	
U-CD vs. HC	Stool	GC-MS	*p*-Cresol, acetone, sulcatone, 2-methyl-butanal, 3-methyl-butanal, carbon disulfide, dimethyl trisulfide, acetic, butyric, valeric acids	Dimethyl trisulfide, dimethyl disulfide, ethyl-acetate, octyl-acetate, 3-methyl-2-oxobutanoic acid, propyl-butanoate, propyl-propanoate, butyl-2-methylbutanoate, glucose, glutamine, Crn	Di Cagno et al., 2009
U-CD vs. HC	Serum	GC-MS	Palmitic, palmitoelic, stearic, oleic acids	Linoleic, alpha-linoleic, arachidonic, eicosapentaenoic, docosapentanoic, docosahexanoic acids	Solakivi et al., 2009
U-CD vs. HC	Serum	H-NMR	Glucose, 3-hydroxybutanoic acid	Lipids, pyruvate, glycoproteins	Bertini et al., 2009
	Urine		IS, Cho, acetoacetate, acetic, propanoic acids		
U-CD vs. HC	Serum	Spectrophotometer-based assay		Total cholesterol	Lewis et al., 2009
U-CD vs. HC	Stool	GC-MS	Iso-caproic, iso-valeric, iso-butyric, acetic acids		Tjellström et al., 2010
U-CD vs. HC	Urine	Spectrophotometer-based assay	NO2, NO3		Högberg et al., 2011
U-CD vs. HC	Serum	H-NMR	Glucose, 3-hydroxybutanoic acid	Lipids, pyruvate, glycoproteins, Cho, Crn, iso-Leu, Leu, Met, Val	Bernini et al., 2011
	Urine		IS, Cho, acetoacetate, acetic, propanoic acids		
U-CD vs. HC	Serum	H-NMR		Lactic acid, valine, lipids	Fathi et al., 2013
U-CD vs. HC	Stool	GC-MS	Acetic, propionic, butyric acids		Caminero et al., 2015
U-CD vs. HC	Plasma	H-NMR	Ala, Gly, Ace, Cr	Crn	Upadhyay et al., 2015
U-CD vs. HC	Stool	HPLC-diode-array detection	Acetic, propionic acids		Primec et al., 2016
U-CD vs. HC	Stool	GC-MS	**	**	Niccolai et al., 2019
U-CD vs. HC	Stool	GC-MS	Acetic acid	Free sulfides, ammonia, L-lactic, propionic, butyric, valeric acids	Zafeiropoulou et al., 2020
U-CD vs. HC	Plasma	H-NMR	Pro, Gly, Arg, Ala, Glu, Cr, Crn, Cys, glucose, lactate, acetate, acetoacetate, β-hydroxybutyrate, pyruvate, succinate, citrate, phosphocreatine	Cho	Upadhyay et al., 2020
	Urine		Pro, Trp, β-hydroxybutyrate, pyruvate, succinate, allantoin, aminohippurate	N-methylnicotinamide	
U-CD vs. HC	Stool	HPLC-MS	Xanthurenic, kynurenic acids	Trp, tryptamine, indole-3-aldehyde, indole-3-lactic acid	Lamas et al., 2020
U-CD vs. HC	Sera	GC-MS	Butyric, iso-valeric, butyric (2-methyl), heptanoic, dodecanoic, tetradecanoic, hexadecanoic, octadecanoic acids	Acetic, propionic, valeric acids	Baldi et al., 2021
U-CD vs. HC	Serum	HPLC-MS		HDL-cholesterol, apolipoprotein-AI, retinol	Jamnik et al., 2018
**Short-term GFD**					
U-CD vs. T-CD	Serum	GC-FID		Steric, arachidonic, docosapentanoic, docosahexanoic acids	Solakivi et al., 2009
U-CD vs. T-CD	Stool	H-NMR		Butyric, valeric, acetic, propionic acids	Tjellström et al., 2014
U-CD vs. T-CD (oat-based GFD)	Stool	H-NMR		Butyric, valeric, acetic, propionic acids	Tjellström et al., 2014
**Long-term GFD**					
T-CD vs. U-CD	Serum	Spectrophotometer-based assay		HDL-cholesterol	Lewis et al., 2009
T-CD vs. U-CD	Serum	Spectrophotometer-based assay		HDL-cholesterol	De Marchi et al., 2013
T-CD vs. U-CD	Serum	Spectrophotometer-based assay		Vitamins (A, B12, D, E), zinc, ferritin, iron	Deora et al., 2017
T-CD vs. U-CD	Stool	HPLC-MS		Indole-3-acetic acid	Lamas et al., 2020
T-CD vs. HC	Stool	GC-MS	Propanone, butyric, valeric acids	Total esters, iso-valeric acid	Di Cagno et al., 2009
T-CD vs. HC	Serum	GC-MS	Palmitic, palmitoleic, stearic, oleic acids	Linoleic, alpha-linoleic, arachidonic, eicosapentaenoic, docosapentanoic, docosahexanoic acids	Solakivi et al., 2009
T-CD vs. HC	Stool	H-NMR GC-MS	Carbon disulfides, 1-octen-3-ol, ethanol and 1-propanol, acetic acids	Ethyl-acetate, octyl-acetate, propyl-butyrate, propyl-propanoate, butyl 2-methylbutanoate, iso-caproic, butyric, propanoic acids	Di Cagno et al., 2011
	Urine		Dimethyl disulfide, dimethyl trisulfide, Lys, Arg, Cr, methylamine	3-Methyl-2-oxobutanoic acid, Crn, glucose, glutamine	
T-CD vs. HC	Serum	H-NMR	Cho	Lipids (mainly VLDL), lactate	Rezaei-Tavirani et al., 2012
T-CD vs. HC	Serum	GC-MS	Valeric acid	Acetic acid	Jakobsdottir et al., 2013
T-CD vs. HC	Saliva	GC-MS	Acetic acid, ethyl ester, nonanal, 1-chlorodecane, trichloromethane, carbon disulfide	2-Ethyl-1-hexanol, 4-(1,1,3,3-tetramethylbutyl)-phenol, ethyl alcohol, butanoic acid 2-methyloctyl ester, thiophenes, ketones, terpenes, 1-octadecene, octanal	Francavilla et al., 2014
T-CD vs. HC	Stool	***		Propionic, butyric, valeric, iso-butyric, iso-valeric acids	Makinder et al., 2014
T-CD vs. HC	Stool	GC-MS	**	**	Rouvroye et al., 2019
T-CD vs. HC	Plasma	LC-MS		Cysteine, cystathionine	Martín-Masot et al., 2020
T-CD vs. HC	Stool	HPLC-MS		Trp	Lamas et al., 2020
T-CD (semi-strict GFD) vs. HC	Serum	Spectrophotometer-based assay	NO2, NO3, NOx		Uspenskaya et al., 2014
T-CD (semi-strict GFD) vs. T-CD (strict GFD)	Serum	Spectrophotometer-based assay	NO3, NOx		Uspenskaya et al., 2014
	Urine			D-xylose	
CD infants (weaned-GFD) vs. CD infants (weaned-GCD)	Stool	H-NMR	**	**	Sellitto et al., 2012
CD-infants vs. HC infants	Serum	HPLC-MS	**	**	Kirchberg et al., 2016 ^#^
**Infants (enrolled before gluten exposure**)					
U-CD infants vs. HC infants	Serum	UHPLC-MS	Lyso-phosphatidylcholine, alkylacyl-phosphatidylcholine, phosphatidylserine	Phosphatidyletanolamine phosphatidylglycerol	Auricchio et al., 2019 ^#^
U-CD infants vs. HC infants	Plasma	UHPLC-MS	Triacylglycerols of low carbon number and double bond count	Phosphatidylcholines	Sen et al., 2019 ^#^
U-CD infants vs. T-CD infants	Plasma	UHPLC-MS	Triacylglycerols (essential and nonessential)	Phosphatidylcholines	Sen et al., 2019 ^#^

For each raw, by referring to the first group reported in the first column, ↑ and ↓ mean a significant increase or decrease, respectively. **No discriminant analyte or significant alteration between groups. ***No methods described. ^#^Longitudinal study. GFD, gluten-free diet; U-CD, active celiac disease subjects; T-CD, GFD-treated celiac disease subjects; HC, healthy controls; GCD, gluten contained diet; GC, gas chromatography; MS, mass spectrometry; H-NMR, nuclear magnetic resonance spectroscopy; LC, liquid chromatography; (U)HPLC, (ultra)high-performance liquid chromatography; Ala, alanine; Arg, arginine; Cho, choline; Cr, creatine; Crn, creatinine; Crn, carnosine; Glu, glutamic acid; Gly, glycine; IS, indoxyl sulfate; Leu, leucine; NOx, nitric oxides; Pro, proline; Trp, tryptophane.

### Outcomes

The primary outcome was to provide an overview regarding the changes in metabolome at the time of CD diagnosis and during its treatment with GFD, based on a comparison against healthy controls (HC). Additionally, a secondary purpose of the present work was to identify some potential metabolites suitable to be used as CD biomarkers.

## Results

According to the fact that gluten constitutes an essential dietary component in Western societies, a strict lifelong GFD leads to the diversity and abundance of gut microbiota. Noteworthy, nutritional regimens, including GFD, involve the production of specific microbial and human metabolites (Bascuñán et al., 2020). In the selected studies, investigators applied different techniques to describe metabolic variations, such as gas- or liquid- chromatography (GC and LC, respectively) coupled with mass spectrometry (-MS), or proton nuclear magnetic resonance (H-NMR) spectroscopy. Besides, carbon source evaluation systems and spectrometer-based assays have been also adopted in a few selected studies.

### Metabolome in Untreated-Gluten-Free Diet Celiac Disease Subjects

In Niccolai et al. (2019), FFAs were profiled in fecal samples of untreated-GFD CD patients (U-CD) and HC. Based on GC–MS analysis, no significant differences emerged allowing the authors to conclude that short-chain fatty acids (SCFAs) in stools did not discriminate between enrolled groups. Subsequently, serum from both groups previously enrolled (Niccolai et al., 2019) was processed by the same research group (Baldi et al., 2021). Following the same GC–MS-based methodological approach, the authors found that U-CD patients had a lower relative concentration of circulating acetic, propionic, and valeric acids than HC. Conversely, a great relative concentration of butyrate and other SCFAs (i.e., *iso*-valeric, 2-methylbutyric, heptanoic, dodecanoic, tetradecanoic, hexadecanoic, and octadecanoic acids), medium chain-FAs, and long chain-FAs were found in U-CD. Considering results from both studies (Niccolai et al., 2019; Baldi et al., 2021), authors suggested that FFA profiles differed in U-CD compared to HC although this was mainly detectable in serum rather than feces.

Among the 33 selected research articles, 6 studies evaluated SCFA in U-CD. Of these, only one (Zafeiropoulou et al., 2020) was in line with Niccolai et al. (2019). With specific regard to the cross-sectional part of this work, no differences in total and single SCFA-compounds in feces of U-CD vs. HC analyzed by GC–MS were found. Instead, a lower concentration of free sulfide and L-lactic acid characterized U-CD than HC fecal samples. An absence of differences in the total SCFAs values was also found by Primec et al. (2016) when processing stools delivered by U-CD and HC through the application of HPLC. Besides, the latter profile revealed a higher concentration of acetic and propionic acids in U-CD than in HC.

Differently, three studies (Tjellström et al., 2010; Nistal et al., 2012; Caminero et al., 2015), applying GC–MS to feces delivered by U-CD and HC, found total SCFAs significantly increased in the first. At the single compound level, the total SCFA increase was determined by a cumulative effect of acetic, propionic, and butyric acids in two out of three studies (Nistal et al., 2012; Caminero et al., 2015). Instead, Tjellström et al. (2010) found that only acetic acid, with a slight contribution of *iso*-caproic acid, determined the increase of the related chemical class.

In complete contrast with this scenario, the study conducted by Di Cagno et al. (2009) found lower total SCFAs in feces delivered by U-CD than HC as a result of GC–MS profiling. Differences in single metabolites were also found, specifically acetic, valeric, and *iso*-valeric acids. Additionally, the authors assessed other chemical classes that were higher in U-CD than HC (i.e., ketones, alcohols and phenols, sulfur compounds, aldehydes, and hydrocarbons). More in-depth, U-CD had a higher relative concentration of *p*-cresol (threefold), acetone (twofold), sulcatone (twofold), 2-methyl- and 3-methyl-butanal (both approximately threefold), carbon disulfide (1.5-fold), and dimethyl trisulfide (threefold) compared to HC.

Lamas et al. (2020) analyzed feces collected from U-CD and HC by applying an HPLC–MS-based investigative approach. Compared to HC, U-CD had lower fecal concentrations of specific metabolites working as activators of the aryl hydrocarbon receptor (AhR), specifically, tryptamine, indole-3-aldehyde, and indole-3-lactic acid. Moreover, the authors assessed a lower concentration of tryptophane (Trp) in feces of U-CD than that of HC while there were higher Trp-derivatives in the first group (i.e., xanthurenic and kynurenic acids).

Upadhyay et al. (2015) investigated blood plasma metabolites of U-CD through H-NMR spectroscopy. A significantly higher concentration of alanine, glycine, acetate, and creatine was found in U-CD samples than those of HC, whereas the creatinine was significantly lower. In a subsequent protocol, as before based on H-NMR spectroscopy, metabolites were extracted from the intestinal mucosa, blood plasma, and urine of U-CD patients and compared against two different control groups (Upadhyay et al., 2020). In detail, HC were used to compare blood plasma and urine, while a cohort of patients affected by gastroesophageal reflux and functional dyspepsia was used as the disease control group (DCG) aiming to compare biopsies. Concerning this last comparison, U-CD patients showed a lower concentration of proline and allantoin, as well as higher concentration of glycine, histidine, and glycerophosphocholine than DCG. Compared to HC, the metabolome of U-CD differed for 18 plasma metabolites and 8 urinary metabolites (see Table 1). All these metabolites were significantly higher in U-CD, except for choline (in blood plasma) and *N*-methylnicotinamide (in urines).

Only a few significant differences were shared combining the results collected by the same research group (Bertini et al., 2009; Bernini et al., 2011), which adopted the same methodological approach based on H-NMR to profile U-CD serum and urine samples. In U-CD sera, lactate, lipids, pyruvate, and glycoproteins resulted lower than HC. Conversely, glucose and 3-hydroxybutanoic acid levels were found higher in sera of U-CD than in the sera of control subjects. Additionally, in sera of U-CD patients, Bernini et al. (2011) detected lower levels of specific amino acids (i.e., choline, creatinine, isoleucine, leucine, methionine, and valine) compared to HC. Shared features from urine samples of these two studies (Bertini et al., 2009; Bernini et al., 2011) concerning levels of indoxyl sulfate (IS), choline, acetoacetate, acetic acid [2-(2-phenylacetyl)-amino-], and propanoic acid [3-hydroxy-3-(3-hydroxyphenyl)-], which were all higher in U-CD than in HC.

Following these intriguing results (Bertini et al., 2009; Bernini et al., 2011), further two studies (Rezaei-Tavirani et al., 2012; Fathi et al., 2013) collected blood samples from U-CD and HC and processed sera through H-NMR spectroscopy. Both the studies mainly discussed the clustering method based on those metabolites that better differentiated U-CD from HC. In the first (Rezaei-Tavirani et al., 2012), the lower levels of lipids (mainly VLDL) and lactate, as well as the higher level of choline, found in U-CD than in HC allowed the authors to differentiate the groups. Instead, the latter work (Fathi et al., 2013) proposed a clustering of samples accounting for 89% accuracy using serum values of total lipids, lactate, and valine, which were found to be lower (*p* < 0.001) in U-CD than in HC.

Based on those findings showing that U-CD had a high level of lipids, Solakivi et al. (2009) more in-depth evaluated the FA profile of U-CD. The lipidomics based on GC-flame ionization detector (FID) assessed higher ratios of palmitic, palmitoleic, stearic, and oleic acids, as well as lower ratios of linoleic, alpha-linolenic, dihomo-gamma-linolenic, arachidonic, eicosapentaenoic, docosapentaenoic, and docosahexaenoic acids than those of HC. Instead, Jamnik et al. (2018) deeply profiled biochemical biomarkers of cardiometabolic health and nutritional status in blood. Compared to those with negative CD serology, U-CD had a lower level of circulating HDL-cholesterol and apolipoprotein-A1. Also, the ratio of total cholesterol to HDL-cholesterol was significantly affected being higher in U-CD than in those with negative CD serology. Concerning markers used to profile the nutritional status, only retinol was significantly reduced in U-CD in fully adjusted models.

Only Högberg et al. (2011) profiled urine intending to deeply investigate the level of nitric oxide products (NOx). Following a large screening for CD, U-CD had significantly increased urinary NOx concentrations compared to HC (Högberg et al., 2011).

### Metabolome in Gluten-Free Diet-Treated Celiac Disease Subjects

Contextually to CD genetics, a long-life exclusion of gluten from the diet is nowadays the only effective treatment in alleviating disorders and symptoms of CD. Following the scopes of the present review, CD individuals under GFD were considered as ongoing-treated patients (T-CD). However, it is important to emphasize that GFD itself impacts gut microbiota composition (Girbovan et al., 2017) and that GFD is not standardized among countries being influenced by different dietary habits. Additionally, the degree of GFD adherence and the duration of treatment can lead to the abundance of specific gut bacterial patterns and, therefore, the gut microbiota metabolism as an adaptive response. Furthermore, in long-term GFD, results derived from metabolomics encompass two different features that need to be considered, which are the restored integrity of the epithelial layer and a more stable gut microbial community, no longer perturbed by the dietary change (Caio et al., 2020). In accordance with this, selected studies have been included in this study in separate sections aiming to differentiate short-term to long-term GFD treatments.

#### Metabolome in Celiac Disease at Diagnosis (U-CD) Short-Term (Maximum 12 Months)-Gluten-Free Diet Treated

As a part of their study, Bertini et al. (2009) analyzed blood and urine samples of newly diagnosed CD (U-CD) and after 3, 6, and 12 months of GFD. After 12 months, serum spectra of T-CD showed decreased levels of glucose and increased lipoproteins. Furthermore, T-CD showed a decrease in 3-hydroxybutyric acid and an increase in some amino acids (asparagine, choline, isoleucine, leucine, methionine, and valine), lactate, and creatinine.

Two further studies screened the levels of serum biochemical parameters in subjects with U-CD who undertook GFD (Lewis et al., 2009; De Marchi et al., 2013). These studies differed for the duration of the follow-up after the dietary change to GFD. Despite this difference, shared findings have been assessed. Both studies notified a significant increase in HDL-cholesterol and a decrease in the ratio of total cholesterol to HDL-cholesterol, after 6–8 months (De Marchi et al., 2013) or 12 months (Lewis et al., 2009). Meanwhile, De Marchi et al. (2013) found a significant increase in total cholesterol in T-CD.

Zafeiropoulou et al. (2020) profiled SCFAs, ammonia, sulfides (free and total), lactic acid (D-isoform, L-isoform, and total) in feces delivered by U-CD that, after diagnosis, undertook GFD. Two follow-ups, specifically at 6 and 12 months, were included in the study design. When analyzed as absolute concentrations, the baseline values of all screened metabolites did not reach significance compared to both follow-ups. Differently, when SCFAs were statistically compared as relative concentration (percentage computed out of the total), authors observed at 6 months of gluten deprivation an increase in acetic acid and the decrease of propionic, butyric, valeric, *iso*-butyric, and isovaleric acid. Among these differences, only the significance related to *iso*-butyric acid extended to 12 months. Similarly, Makinder et al. (2014) enrolled U-CD that provided feces after 6 and 12 months of GFD, but in this case the dietary gluten deprivation did not determine changes in levels of SCFAs. Instead, the authors noticed a significant increase in fecal sulfide after both 6 and 12 months on GFD (Makinder et al., 2014).

The effect of different GFD styles was investigated by Tjellström et al. (2014). Authors compared baseline values (CD-diagnosis) to those collected in T-CD under standard GFD (std-GFD) and to T-CD that followed an oat-based GFD (oat-GFD). Volunteers provided fecal samples after 6 and 12 months of both GFDs. Compared to baseline, no differences in concentration of single SCFA were found at each follow-up (6 and 12 months) in both the study arms (std-GFD and oat-GFD). Nonetheless, the arm of std-GFD showed a significant decrease in the total class of SCFAs after 12 months of diet. Instead, in oat-GFD arm, the total SCFAs did not differ during the trial.

#### Metabolome in Long-Term (at Least 12 Months)-Treated Celiac Disease (T-CD)

To validate metabolites affected by long-term GFD, Di Cagno et al. (2009, 2011), profiled T-CD children two times applying untargeted metabolomics. In the first, the authors collected feces from T-CD and their healthy siblings (HS) using the latter as controls (Di Cagno et al., 2009). Compared to HS, authors found total ketones significantly higher in T-CD (particularly propanone, over threefold), while total esters were reduced significantly. No difference was found in total SCFAs between T-CD and HS, although butyric and valeric acids were higher in T-CD than HS while *iso*-valeric acid showed the opposite. In the successive work, authors profiled through two untargeted metabolomic methods (GC-MS and H-NMR) feces and urine of T-CD and HC (Di Cagno et al., 2011). In feces, T-CD had a higher concentration of total alcohols. Ketones, aldehydes, hydrocarbons, and aromatic heterocyclic compounds were lower in T-CD than HC while, as before, no difference emerged with respect to the relative concentration of total SCFAs. In urine, dimethyl disulfide and dimethyl trisulfide (among VOCs) and lysine, arginine, creatine, and methylamine (by H-NMR analysis) mainly characterized T-CD. The opposite concerned carnosine, glucose, and glutamine (as H-NMR results) and the 3-methyl-2-oxobutanoic acid (VOC).

To cross-sectionally compare metabolites of T-CD, Zafeiropoulou et al. (2020) enrolled HS as well as U-CD and HC. No difference emerged from the comparison of T-CD and HS. However, some fecal metabolites significantly characterized T-CD, such as a lower fecal concentration of ammonia (vs. HC and U-CD) and D-lactic acid (vs. U-CD) and a higher concentration L-lactic acid (vs. U-CD). Also, Makinder et al.’s (2014) study, the concentration of total SCFA and branched-chain fatty acids (BCFAs) did not differ between T-CD and HS, whereas propionic, butyric, and valeric acids as well as *iso*-butyrate, *iso*-valeric, and *iso*-caproic were significantly lower in the same T-CD than HC. In T-CD, the authors also assessed a higher value of total sulfide than HC.

Conversely to the four earlier cited studies (Di Cagno et al., 2009, 2011; Makinder et al., 2014; Zafeiropoulou et al., 2020), Nistal et al. (2012) showed that T-CD had the highest concentration of fecal SCFAs. More in-depth, this class of FAs was markedly affected by the higher levels of acetic, propionic, and butyric acids compared to HC under a gluten-containing diet. To a better understanding, HC undertook GFD for 1 week and, based on further profiling, authors confirmed that SCFAs characterized T-CD independently of gluten deprivation. A similar study was then designed by Caminero et al. (2015) aiming to assess the levels of SCFAs in T-CD, in HC under gluten-containing diet, and HC that undertook GFD for 1 week. As above, due to a higher absolute concentration of acetic, propionic, butyric, and valeric acids, the chemical class of SCFAs was significantly higher in T-CD than in both (gluten-containing and GFD) the HC groups.

Rouvroye et al. (2019) profiled volatile compounds in stools of T-CD and HC adopting a GC coupled to an ion mobility spectrometer (IMS) and assessed that T-CD spectra significantly differed from those of HC. Moreover, the collected spectrum profiles, the level of sensibility, and the level of specificity strongly discriminate samples belonging to T-CD from those delivered by HC. However, neither ion spectra were further processed, nor information was provided on those compounds that significantly differentiated the groups.

Fecal metabolites of T-CD were compared against both U-CD and HC by Lamas et al. (2020). Trp concentration was lower in T-CD than in HC. Moreover, mainly focusing on metabolites involved in the aryl-activation, the authors found no difference in fecal concentrations of Trp derivatives recognized to activate the AhR signaling (i.e., tryptamine, indole-3-aldehyde, and indole-3-lactic acid) between T-CD and U-CD. Instead, the significance was reached by comparing the fecal concentration of indole-3-acetic acid, an additional AhR agonist, which was higher in T-CD and in U-CD.

Solakivi et al. (2009) profiled blood FAs in T-CD adopting GC–FID-based lipidomics. The comparison of U-CD with T-CD determined that among polyunsaturated FAs (PUFA), arachidonic, docosapentaenoic, and docosahexaenoic acids significantly increased in T-CD. The same tendency was assessed for stearic acid. However, despite the collected increase of the above-mentioned lipids during remission (CD under GFD), these remained lower in T-CD than in the HC. Although no mention about the time of adherence to GFD was specified, Jakobsdottir et al. (2013) evaluated SCFAs in blood by GC–MS concluding that no difference was observed in the total amount of SCFAs between T-CD and HC.

The metabolomic signature in plasma was also investigated by Martín-Masot et al. (2020) comparing T-CD to HS. Based on LC-MS/MS analysis, the authors mainly focused on the one-carbon metabolism. Differences in spectra of T-CD blood compared to those of their HS were poorly detectable. Only cysteine and cystathionine were lower in plasma of T-CD than HS and for this reason, the authors suggested a specific defect of the related enzymes, specifically cystathionine beta-synthase (EC 4.2.1.22) and cystathionase (EC 4.4.1.1), that GFD was not able to fill.

Aiming at assessing the nutritional improvement after the U-CD patients undertook GFD, serum metabolites were profiled by Deora et al. (2017). No controls were enrolled. At diagnosis, vitamin D was the most commonly deficient micronutrient. In addition, plasma ferritin, iron, selenium, as well as vitamins A, E, and B12 were significantly deficient. In T-CD cases, who were strictly adherent to GFD, only 2 micronutrients, specifically vitamin D and ferritin, continued to be at suboptimal level after 18 months of GFD.

Serum samples are useful also to screen levels of NOx, as purposed by Uspenskaya et al. (2014). The authors determined that higher values of NO_2_, NO_3_, and total NOx discriminate U-CD (then following GFD) to HC. Besides, this study distinguished T-CD in those strictly adherent to GFD to those who were not strictly adherent to GFD. Compared to HC, T-CD on a strict GFD had no significant differences in all NOx subtype levels. Instead, patients on a semi-strict GFD showed a higher median value of NO_3_ and NOx than HC and T-CD both on a strict GFD.

As part of the non-conventional field of evaluation, the case–control study designed by Francavilla et al. (2014) profiled the salivary metabolome of T-CD. Compared to HC, various metabolites significantly differed in T-CD. The median value of total alcohols (and phenols) was significantly lower in T-CD compared with HC. Both total esters and total sulfur compounds mainly characterized T-CD. Furthermore, the authors evaluated the catabolic profile of oral microbiota assessing that the substrate utilization pattern (H’ index) and substrate richness (S index) were significantly decreased in the saliva of T-CD children compared with HC.

### Metabolome in Infants With Celiac Disease Weaned With or Without Gluten

Four studies collected biological samples from infants screened for CD predisposition. Compared to profiles obtained at the time of positive diagnosis of CD (presence of antibodies and intestinal biopsy proven), authors longitudinally evaluated metabolic differences based on gluten introduction during weaning.

Sellitto et al. (2012) collected feces of infants from birth to 24 months of age aiming to evaluate differences determined by an early gluten introduction during the weaning in infants with CD predisposition. Multiple fecal samples have been collected at different time points from three infants with delayed gluten exposure (simulating GFD) and further three infants belonging to the gluten-containing diet group. Fecal metabolomics was carried out by H-NMR spectroscopy. Fecal samples were clustered according to the age of the children. Differently, early- or delayed-gluten exposure did not discriminate between samples. Based on the first detection of antibodies, a deeper investigation was conducted on the only infant who developed CD at 24 months of age. Fecal samples collected from this subject at the 6th and 8th month of age were significantly different from samples of other pair-aged infants due to high levels of lactate that, only in this subject, continued to be higher than paired-aged infants up to 12 months of age. Also, Kirchberg et al. (2016) investigated the metabolomes in 4-month-old infants from a large cohort composed of 230 subjects of whom 33 showed CD later in life (median age of diagnosis: 3.4 years). Based on adjusted *p*, no discriminant analyte or pattern was found between U-CD compared with others.

Two studies deeply explored serum lipidomics in infants pre- and post-weaning (Auricchio et al., 2019; Sen et al., 2019). Both carried out an ultra-high performance (UHP)-LC-MS analysis, and both included HC to define differences against CD. Auricchio et al. (2019) analyzed samples at 4 months (pre-gluten exposure), 12 months (post-gluten exposure), and at diagnosis of CD (>24 months). Children defined as HC means that they did not develop CD up to 8 years of age. No class of lipids showed significant differences between these groups comparing samples delivered at 4 and 12 months of age. Therefore, multivariate models were then adopted by authors to reduce confounding factors and exacerbate the presence of those metabolites that could be able to discriminate CD from HC. This strategy allowed the authors to obtain, already in samples delivered at the 4th month, the best clustering (100% of CD and about the 66% of HC) when a subset of 9 lipids out of 327 identified was used.

Due to a large number of collected metabolites, even Sen et al. (2019) evaluated differences thanks to a multivariate approach. Reducing confounding variables, the authors mainly assessed that non-essential triacylglycerols were upregulated in CD-progressor infants before the first gluten exposure and that this tendency was reverted after the dietary change to GFD.

## Discussion

Advancements in the field of microbiota (and microbiome) characterization by means of different meta-omics approaches are continuously increasing. The literature widely supports the hypothesis that any change in dietary habits shapes the gut microbial community (Moschen et al., 2012; Senghor et al., 2018; De Angelis et al., 2020). In turn, the composition and function of gut microbiota play a pivotal role in chronic inflammatory diseases (de Vos and de Vos, 2012). Although based on the host genetic background, CD needs to be included among inflammatory diseases due to its peculiar dietary-based influence.

**FIGURE 2:**
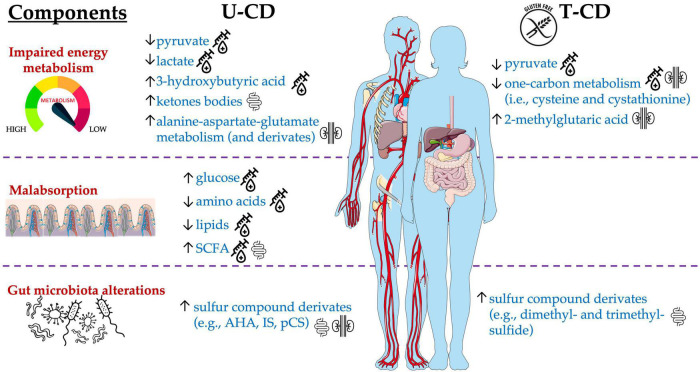
Main metabolites significantly altered in T-CD compared with U-CD, detected in blood, urine, and stool samples.

According to previous evidence (Ryan et al., 2015), the metabolomic signature of CD encompassed three components: impaired energy metabolism, malabsorption, and gut microbiota alterations (Figure 2). Serum analyses from U-CD showed an increased concentration of glucose (Bertini et al., 2009; Bernini et al., 2011; Upadhyay et al., 2020) but reduced levels of pyruvate and lactate (Bertini et al., 2009; Fathi et al., 2013). This energetic impairment directly impacts the weakness and the chronic fatigue reported by U-CD at diagnosis (Jelsness-Jørgensen et al., 2018) and opens the way to another signature commonly observed in selected studies, that is, the high synthesis/utilization of lipids and ketone bodies as the energy sources instead of glucose. Some of the selected studies assessed higher levels of ketogenic metabolites in U-CD than in HC (Bertini et al., 2009; Di Cagno et al., 2009; Bernini et al., 2011; Upadhyay et al., 2020). Similarly, Bertini et al. (2009) analyzed the same subjects pre- and post-GFD and observed an inverse tendency in serum levels of β-hydroxybutyric acid, which is a ketone body usually increased during the fasting phase as an alternative energy source for brain activity (Ota et al., 2021). Hence, the significant decrease of β-hydroxybutyric acid (Bertini et al., 2009) seems to indicate the return to a normal energy metabolism *via* carbohydrates instead of ketone bodies driven by GFD.

**FIGURE 3:**
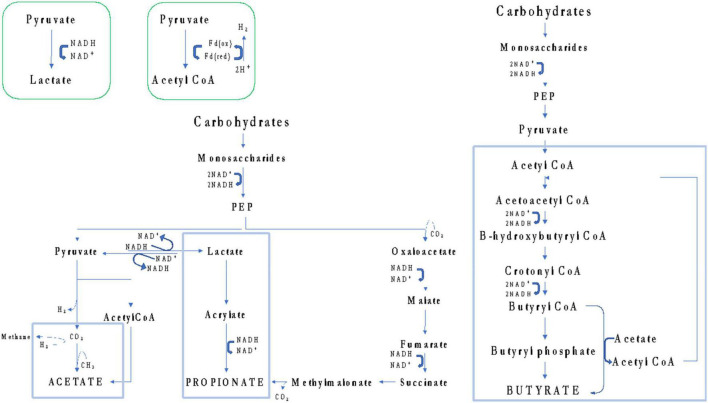
Schematic overview of pathways that gut microbes can use to synthesize acetate, propionate, and butyrate from carbohydrates.

Among selected articles, the authors also evaluated the energy balancing through SCFA profiling. Di Cagno et al. (2009) found a critical lower concentration of total SCFAs in U-CD when compared to HC, while subjects who undertook GFD (T-CD) did not exhibit the same behavior. Contrarily, other studies reported the complete absence of differences in the levels of total SCFAs (Primec et al., 2016; Niccolai et al., 2019; Zafeiropoulou et al., 2020) or their increase (Tjellström et al., 2010; Nistal et al., 2012; Caminero et al., 2015; Baldi et al., 2021). The adoption of different methods (GC- or LC-MS), sample type (serum or feces), or data elaboration (absolute or relative concentration) could have led to these controversial results. In addition, the SCFA metabolism is dependent on the availability of SCFA precursors (e.g., glucose, pyruvate, and lactate) but is also influenced by the dietary consumption of prebiotics (Figure 3). In turn, prebiotic consumption drives the abundance of SCFA-producing microbes in the gut (Golfetto et al., 2014; Makki et al., 2018). However, as recently reviewed by Verdu and Schuppan (2021), due to controversial results, the microbiota composition in CD did not show a unique fingerprint. Although some taxa shared common tendencies (i.e., increased abundance of Proteobacteria, *Neisseria*, and *Escherichia coli* as well as a decreased abundance of *Bifidobacterium* and *Bacteroides ovatus*), not a unique microbiota fingerprint is consistent with CD. Similarly, a lack of dietary fibers was claimed to characterize GFD (Thompson et al., 2005; Hopman et al., 2006; Martin et al., 2013), although this evidence is highly dependent on dietary habits that, in turn, are mainly influenced by culture and geography (De Angelis et al., 2020).

As for SCFAs, also findings concerning the protein metabolism showed a broad heterogenicity among selected studies. In the studies by Bertini et al. (2009); Bernini et al. (2011), Fathi et al. (2013); Martín-Masot et al. (2020), and Lamas et al. (2020), specific amino acids could be usually detected at low levels in U-CD serum. Oppositely, high concentrations of sulfur compounds and nitrogen derivatives have been found in urine (Bertini et al., 2009; Bernini et al., 2011; Di Cagno et al., 2011; Upadhyay et al., 2020), feces (Di Cagno et al., 2009), and saliva (Francavilla et al., 2014). On one hand, the low levels of amino acids in serum could suggest an impaired host metabolism as well as an unbalanced dietary intake (Martín-Masot et al., 2020). Instead, the collection of high concentrations of IS, *p*-cresol sulfate (pCS), and trimethylamine-*N*-ox (TMAO) require the presence of specific gut microbes able to metabolize the relative precursors (tryptophan or indole for both IS and pCS, while trimethylamine for TMAO). The occurrence of these metabolites allows us in speculating about two additional CD manifestations, which are the altered (reduced) intestinal permeability and the confirmation of high abundance of some specific taxa (e.g., Proteobacteria, *Enterobacteriaceae*, *Escherichia*, and *Klebsiella*) as shown by previous works (D’argenio et al., 2016; Jameson et al., 2016). Additional evidence about the impaired microbiota metabolism in CD comes to light from Bernini and co-workers (2011), who observed that using blind H-NMR-profiled urinary spectra, metabolites derived from microbiota [mainly IS, m-propionic (hydroxyphenyl) acid, and amino-acetic (phenylacetyl) acid] discerned U-CD from T-CD and HC.

About fats, the lipidomics carried out by Solakivi et al. (2009) explained those abnormalities in FA levels characterizing U-CD, as well as the benefits given by the GFD adoption. When evaluating the same individuals after 12 months of GFD, long-chain (C20–22) PUFAs increased although this variation was not sufficient to reach the levels found in HC. Long-chain (C20–22) PUFAs are important for optimal metabolism while deficiencies might contribute to the disease onset, including psychiatric diseases and depression that, indeed, are manifestations usually reported by U-CD (De Palma et al., 2014; Jelsness-Jørgensen et al., 2018). Impairments in FAs determined by CD were also argued by Jamnik et al. (2018). This research group showed that U-CD had a lower level of circulating HDL-cholesterol and apolipoprotein-A1. However, concerning HDL-cholesterol, two studies notified that GFD was able to reverse its levels after 6–8 months (De Marchi et al., 2013) and after 12 months (Lewis et al., 2009).

However, the first goal of GFD undoubtedly remains the improvement of the epithelial integrity, amelioration of the intestinal functionality, and the reduction of the inflammation status. In line with the results collected by Högberg et al. (2011) in urine profiles, also Uspenskaya et al. (2014) found that higher values of NO_2_, NO_3_, and total NOx discriminate serum from U-CD and HC. The assessment of high levels of these compounds suggests an ongoing inflammatory status in U-CD. Compared to HC, T-CD patients following a strict GFD showed no more significant differences in levels of all the NOx subtypes. Instead, in those T-CD that were on a semi-strict GFD (compared both with HC and T-CD on a strict GFD), higher median values of NO_3_ and NOx persisted.

Despite the absence of fully concordant results, some alterations affecting U-CD can be highlighted in this study. The end-products from gluten metabolism activate the immune response in the hosts resulting in the overexpression of different pathways involved in the cascade of inflammation signaling. Already at the level of intestinal epithelial cells, an ongoing inflammation status was emphasized by the high levels of NOx found in serum and urine (Högberg et al., 2011; Uspenskaya et al., 2014). Consequently, many amino acids involved in the proliferation, repair, and healing of cells are altered being found increased or decreased based on their biological function as well as on the degree of inflammation (Upadhyay et al., 2020). For example, as deeply evaluated by Lamas et al. (2020), the Trp-derivatives activating the AhR-pathway were too low in U-CD determining an impairment in the AhR signaling of immune responses at the mucosal barrier site level. Moreover, both the bioavailability and the absorption of specific amino acids can directly affect the glycolysis that, in most U-CD patients, they were down-regulated (high glucose/low pyruvate and lactate) requiring the synthesis of alternative sources of energy (e.g., ketone bodies). The great heterogenicity of data reflects the broad combination of different affecting factors. First, although being under long-term GFD, the degree of gluten deprivation (strict/semi-strict) is widely determinant. In this line, evidence underlines how many patients can be labeled as non-responders to GFD (Kivelä et al., 2021). Furthermore, dietary habits, in terms of macro- and micronutrient intake, are pivotal factors in determining the microbiota fingerprint and the resulting gut metabolome. Therefore, the only assessment of gluten deprivation is not sufficient to stratify T-CD patients within a cohort. According to this consideration, when future metabolomic studies are developed, it may be useful to gather much more information on the type of GFD followed through the determination of macro- and micronutrient intake. Only after stratifying patients according to their dietary intake will the involvement of GFD also be further understood.

Further four studies (Sellitto et al., 2012; Kirchberg et al., 2016; Auricchio et al., 2019; Sen et al., 2019) fully verified our inclusion criteria but, considering their peculiar study designs, need to be separately discussed. Kirchberg et al. (2016) and Sellitto et al. (2012) performed two longitudinal cross-sectional studies enrolling infants. In CD-predisposed infants aged 4, the evaluation of metabolomes was not sufficient to anticipate CD-onset (Kirchberg et al., 2016). For this reason, the authors suggested that metabolomics was poorly predictive in the first years, reflecting that the serum metabolic pathways will be mainly affected only later in life. Due to the age of enrolled infants, also Sellitto et al. (2012) did not report differences in the aforementioned metabolites. The authors found that samples collected during the first 24 months after birth clustered according to age more than based on gluten introduction during weaning (Sellitto et al., 2012). This is in accordance with previous studies on infants, which reported how various prenatal and perinatal factors influence the early gut microbiota development (Koenig et al., 2011; Raspini et al., 2020). In addition, although the feeding and the time of solid food introduction were demonstrated to be among the main postnatal factors affecting gut microbiota in early phases of life (Differding et al., 2020; Raspini et al., 2021; Vacca et al., 2022), the fact that only one infant showed positive antibodies before 24 months of age in Sellitto et al. (2012), in our opinion, was not statistically sufficient to raise conclusions about effects related to an earlier or delayed gluten introduction in infants with genetically predisposed CD. Lipidomes, on the contrary, have provided more information as predictive tools to assess the CD onset later in life, suggesting that specific phosphatidylcholines might differentiate CD infants before gluten exposure (Auricchio et al., 2019). Sen et al. (2019) instead argued how several nonessential triacylglycerols were upregulated in infant CD progressors.

### Future Perspectives

Overall, this review pointed out the microbial involvement in CD patients since the first phases of the gluten-related disorders despite the wide heterogeneity of significant differences that exist between U-CD and HC. Moreover, although GFD is required as nutritional therapy in overt-CD individuals being sufficient to avoid the epithelial disruption relapsing, GFD needs to be implemented in the first instance with dietary fibers. In this line, dietary supplementations were recently verified in different trials (Krupa-Kozak et al., 2017; Feruś et al., 2018; Drabińska et al., 2020), but no findings according to microbiota-related outcomes (thus, neither to relative metabolites) have been disclosed. Additionally, strict adherence to a GFD was difficult to be followed by many patients, and, therefore, small amounts of gluten have been periodically taken leading to microbial (and metabolic) imbalances. Interesting approaches involve the gluten sequestering (e.g., by gliadin-targeting antibodies or polymeric binders) before it is metabolized into immunogenic peptides (McCarville et al., 2014; Sample et al., 2017). Instead, enzyme-based therapies are investigated as innovative approaches aimed at inactivating immunogenic gluten peptides by peptidase supplementation. At the same time, any peptidase can completely degrade both gluten and immunogenic derivatives. Being a natural source of peptidases, microorganisms (both bacteria and fungi) can contribute to digest peptides. However, not a unique strain possesses the entire peptidase pattern needed for peptide hydrolyzation. Hence, the definition of a cocktail of peptidases is essential for preliminary research activity. In line with this, recent efforts are working out to develop GFD-complements useful in preventing accidentally ingested small amounts of gluten (Herrán et al., 2017; Cavaletti et al., 2019; De Angelis et al., 2021). A deeper understanding of the relationship occurring between the intestinal microbiota metabolism and the immune response may represent a useful research topic in the context of new biomarkers for CD diagnosis. To date, probiotics have been tested to evaluate their postbiotic contributions to enhancing the intestinal eubiosis and well-being in CD (Francavilla et al., 2020) but previous works (Smecuol et al., 2013; Quagliariello et al., 2016; Francavilla et al., 2019; Håkansson et al., 2019) focused only on immunity outcomes, proinflammatory cytokines, and gut microbial variations, without investigating the metabolomic area.

## Conclusion

Celiac disease is a condition involving the host’s genetics and environment. The current literature widely supports the hypothesis that perturbations of the intestinal homeostasis exacerbate the CD symptomology through different mechanisms, and, in this context, the microbiome structure and function markedly contribute. Metabolomics gives the opportunity of studying the results coming from the interaction between host, diet, and microbiota, highlighting variations in healthy and diseased states. However, currently, it is not possible to give a unique statement about a metabolomic fingerprint related to CD because of the specific influence of diet, host genetics, disease grade, race or age, microbiome, and methodological approaches. The great heterogenicity of study designs, applied methodological approaches, and the limited number of research articles precludes the possibility to conclude. *Ad hoc* prepared procedures and databases collecting data and related metadata, together with standardized analysis protocols, are required. Therefore, even if there is a growing interest in exploring the factors triggering CD through meta-omics approaches, the prospective to having a CD diagnosis by applying a metabolomic non-invasive investigation is still very far away also considering the multiple expression forms of gluten-related disorders.

## Author Contributions

MV and FMC: conceptualization. AP, MV, TL, and II: investigation. MV, FMC, AP, and GC: writing—original draft preparation. All authors contributed to writing—review and editing. All authors have read and agreed to the final version of the manuscript.

## Conflict of Interest

DP was employed by the Giuliani SpA. The remaining authors declare that the research was conducted in the absence of any commercial or financial relationships that could be construed as a potential conflict of interest. The handling editor declared a past co-authorship with several authors, MV, FMC, and MD.

## Publisher’s Note

All claims expressed in this article are solely those of the authors and do not necessarily represent those of their affiliated organizations, or those of the publisher, the editors and the reviewers. Any product that may be evaluated in this article, or claim that may be made by its manufacturer, is not guaranteed or endorsed by the publisher.
